# Association between the Planetary Health Diet Index and growth differentiation factor-15: the Seniors ENRICA-2 cohort

**DOI:** 10.1007/s11357-025-01712-8

**Published:** 2025-05-31

**Authors:** María del Carmen Aznar de la Riera, Rosario Ortolá, Blanca Fabre-Estremera, Antonio Buño-Soto, Fernando Rodríguez-Artalejo, Mercedes Sotos-Prieto

**Affiliations:** 1 Department of Preventive Medicine and Public Health, School of Medicine, Universidad Autónoma de Madrid, Avda del Arzobispo Morcillo, 4, 28029 Madrid, Spain; 2 CIBERESP (CIBER of Epidemiology and Public Health), Av. Monforte de Lemos, 3-5. 28029 Madrid, Spain; 3 Department of Laboratory Medicine, La Paz University Hospital, 28046 Madrid, Spain; 4 IdiPaz-Hospital La Paz Institute for Health Research, Madrid, Spain; 5 IMDEA-Food Institute. CEI UAM+CSIC, Ctra. de Canto Blanco 8, E. 28049, Madrid, Spain; 6 Department of Environmental Health, Harvard T.H. Chan School of Public Health, 665 Huntington Avenue, Boston, MA 02115 USA

**Keywords:** Planetary health diet, Plant-based diets, Cardiovascular disease, Inflammaging biomarkers, Epidemiology

## Abstract

The growth differentiation factor-15 (GDF-15) is an inflammaging biomarker, predictor of cardiovascular disease (CVD), and several other non-communicable diseases (NCD) that represent the main causes of death globally, for which prevention is essential. Current worldwide threats include NCD and environmental burden, where diet is a key determinant. Therefore, the EAT-Lancet Commission developed the Planetary Health Diet Index (PHDI), a dietary pattern designed to ameliorate human and environmental health. The objective of the study was to evaluate the association between the PHDI and serum concentrations of GDF-15 in older adults. Study participants were 2497 people aged + 65 years from the Seniors-ENRICA-2 study. Food consumption was obtained through a validated diet history, and adherence to the PHDI was estimated with a score of 15 food groups (range 0–140), where higher scores indicated better adherence. Analyses for the association between the PHDI and GDF-15 concentrations were performed using multivariable linear regression models, adjusting for main potential confounders. The PHDI showed an inverse dose–response association with GDF-15 concentrations. Compared with participants in the lowest quartile of the PHDI, those in the highest quartile had a mean percentage difference (95% CI) of − 6.8% (− 11.1, − 2.4) in GDF-15 concentrations. The mean percentage difference (95% CI) per 20-point increase of adherence was − 4.4% (− 7.7, − 0.9). Consumption of whole grains, fruits, nuts, and a low intake of trans and saturated fat were important drivers of this association. Results remained robust after adjustment for other inflammation (interleukin 6), renal and cardiac (creatinine, NT-proBNP, cardiac troponin T) biomarkers, and after excluding participants with CVD, type 2 diabetes, and obesity. The PHDI was associated with lower concentrations of GDF-15. Adopting the PHDI may be a useful approach to reduce chronic inflammation and target NCD prevention in the older adult population, while staying within planetary boundaries.

**Supplementary Information:**

The online version contains supplementary material available at 10.1007/s11357-025-01712-8.

## Introduction

Several inflammation biomarkers whose levels increase with age, also known as “inflammaging” markers, have grown in interest given their role as predictors of cardiovascular disease (CVD), several other noncommunicable diseases (NCD), and mortality [1]. One of them, the growth differentiation factor-15 (GDF-15), first known as the macrophage inhibitory cytokine-1 (MIC-1), is released as a response to oxidative stress, inflammation, and tissue injury [1–3]. The modulation of GDF-15 might be key in preventing the development and progression of CVD [1, 2], diabetes [4], or kidney disease [5, 6].

Therefore, different approaches have been designed to target GDF-15 through the adoption of an adequate lifestyle and dietary habits [7–9], given that they may both have direct and indirect (mediated by GDF-15) effects on NCD prevention and management. Previous studies have found that higher adherence to a Mediterranean lifestyle (represented by the MEDLIFE index) [8], the Mediterranean diet [7], an overall healthy diet (as per the Alternate Healthy Eating Index–2010 (AHEI-2010)) [7], and the DASH (Dietary Approach to Stop Hypertension) diet [7] were associated with lower concentrations of GDF-15 in a cohort of older adults. Conversely, a pro-inflammatory diet, assessed with the Dietary Inflammatory Index (DII), has been associated with higher GDF-15 concentrations [7].

However, diet quality is not only important for human health but also has a notorious impact on environmental health [10]. Adopting a “diet–environment–health” trilemma mindset [11] is necessary given the expected increase in NCD, partly resulting from population aging, and the substantial environmental burden of food systems (responsible for up to 30% of global greenhouse gas emissions, and 70% of freshwater use) [10]. The translation towards plant-based diets—characterized by a high intake of healthy plant-based foods and a reduced reliance on animal-sourced products, which are associated with a higher health and environmental burden [10, 12]—could simultaneously address both human and planetary health challenges. In this context, in 2019, the EAT-Lancet Commission defined the Planetary Health Diet (PHD), a dietary pattern characterized by a high intake of plant-based foods and a low consumption of animal-sourced foods [10]. Since then, several studies have found that adherence to the PHD is associated with a lower risk of CVD [13, 14], diabetes [14, 15], and all-cause death [16–18], which represent conditions with an inflammatory basis that have been associated with higher plasma concentrations of GDF-15 [3, 4, 19–21]. Similarly, the Malmö Diet and Cancer cohort [9], the only study that has evaluated the effect of the EAT-Lancet Dietary Pattern on several biomarkers in middle-aged adults observed that GDF-15 was linked to higher risk of heart failure but inversely associated with adherence to the PHD, potentially given the anti-inflammatory properties of this diet. Notably, adherence to the PHD was assessed using a categorized scoring criterion [17], which may not have captured habitual food consumption as accurately as a continuous and prorated score.

To our knowledge, no previous study has evaluated the effect of the PHD on GDF-15 in older adults, who are more prone to age-associated subclinical inflammation and, thus, at higher risk of CVD and other NCDs, the main causes of death worldwide, accounting for over 70% of deaths annually [22]. Moreover, no previous research has investigated this association, regardless of the age of the study population, using the continuous and prorated score developed by Bui et al. [16]. For this reason, and considering the growing aging population [10], for whom healthy aging will play a determinant role in shaping public health strategies, we aimed to evaluate the association between adherence to the PHD and GDF-15 levels in a cohort of older adults.

## Methods

### Study design and participants

Data was obtained from the baseline wave of Seniors-ENRICA (Study on Nutrition and Cardiovascular Risk)−2 cohort [23–25], whose methods have been reported previously. In brief, 3273 participants were selected between 2015 and 2017 by stratified random sampling of community-dwelling adults aged over 65 holding a national healthcare card and living in the metropolitan area of Madrid (Spain) or four surrounding large towns. First, information on sociodemographic, morbidity, and lifestyle data was obtained through a telephone interview. Then, two home visits were conducted to collect biological samples, perform a physical examination, and assess food consumption with a diet history. The average time between the telephone interview and the first home visit was 37 days, and 7 days between the first and the second home visit. The study was approved by the Clinical Research Ethics Committee of “La Paz” University Hospital in Madrid. Written informed consent was given by study participants.

### Study variables

#### The Planetary Health Diet Index

Food consumption was ascertained using a computerized, face-to-face, validated dietary history [26]. This electronic diet history, also known as the ENRICA diet history (DH-E), was developed from the diet history used in the European Prospective Investigation into Cancer and Nutrition (EPIC) cohort study in Spain [25]. The DH-E collects information on 861 foods, including 184 recipes commonly cooked in Spain, and includes 127 sets of digitized photographs to help estimate food portions [26]. Macro- and micro-nutrients were estimated using the standardized food composition tables [26, 27] incorporated in the DH-E.

By utilizing the aforementioned data, adherence to the Planetary Health Diet was estimated by the Planetary Health Diet Index (PHDI), following the criteria defined by Bui et al. [16]. The criteria used to build the PHDI are based on evidence for health outcomes and environmental impact, considering that it would be consumed by the whole world’s population in 2050 (expected to be around 10 billion). In brief, the PHDI was built on 15 food groups: whole grains, starchy vegetables, vegetables, whole fruits, dairy foods, red/processed meat, chicken and other poultry, eggs, fish/shellfish, nuts, non-soy legumes, soybean/soy foods, added saturated and trans-fat, added unsaturated oils, and added sugar and fruit juice. For each food group, a range of potential intakes was specified within a fixed total energy intake (2500 kcal/day), reflecting the core principle of dietary substitution. The least beneficial health effect of each food group (frequently 0 g/day for healthy food groups) received the minimum score (0 points), reflecting minimum daily consumption. In contrast, the maximum score for each food group was 10 points (except for non-soy legumes and soy foods, which was 5) and reflected the maximum daily consumption, the highest beneficial health effect of that group (0 g/day for unhealthy food groups). Intermediate scores were assigned proportionally to consumption levels between minimum and maximum. The total PHDI score ranged from 0 to 140 points (highest adherence to the PHDI) (Supplementary Table S1).

#### GDF-15

Data on serum GDF-15 concentrations were obtained from 12-h fasting blood samples collected from each participant at the first home visit. The samples were gathered in rapid serum tubes with a thrombin-based clot activator and polymer gel (Becton Dickinson). Within 1 h after collecting the tubes, they were centrifuged at 2520 g and room temperature (20–23 °C) for 10 min. Then, serum was aliquoted and frozen at − 80 °C and stored for up to 3.6 years at the Department of Preventive Medicine and Public Health at the Universidad Autónoma de Madrid. Between July 2019 and June 2020, serum GDF-15 was quantified at the Department of Laboratory Medicine of “La Paz” University Hospital by an electrochemiluminescence Elecsys® immunoassay method using a cobas® 6000 analyzer (Roche Diagnostics). For a GDF-15 average concentration of 7343 pg/mL, the interassay coefficient of variation (CV) was 5.4% and 7.7% for an average concentration of 1428 pg/mL.

### Potential confounders

Self-reported sociodemographic variables included sex, age, educational level (primary, secondary, university), tobacco status (current, former, never), and alcohol consumption (never, former, moderate [≤ 10 g/day in women and ≤ 20 g/day in men] [28], or heavy). Regarding other lifestyle variables, body mass index (BMI) was estimated as weight (kg) divided by square height (m^2^), both measured under standardized conditions. Physical activity (metabolic equivalents of task-hour/week-METs [29, 30]) and sedentary behavior, approximated by the time spent watching TV (≤ 2 and > 2 h/day), were also reported by participants. Energy intake (kcal/day) was assessed from dietary history. Regarding medical history, the presence of CVD was assessed by self-reporting a medical diagnosis of myocardial infarction, stroke, or congestive heart failure and type 2 diabetes as a self-reported medical diagnosis, being under antidiabetic medication, or having a fasting blood glucose ≥ 126 mg/dL. Systolic blood pressure was measured three times under standardized conditions and using validated devices, and the average of the second and third measurements was used for analyses. As for clinical biomarkers, 12-h fasting serum glucose, creatinine, total cholesterol, triglycerides, and high-density lipoprotein cholesterol (HDL-C) were measured with colorimetric enzymatic methods using Atellica® Solution CH (Siemens Healthineers). Low-density lipoprotein cholesterol (LDL-C) was calculated with the Friedewald formula (LDL-C = total cholesterol − triglycerides/5 – HDL-C) if triglycerides were < 250 mg/dL and measured by a colorimetric enzymatic method on Atellica® Solution CH if triglycerides were ≥ 250 mg/dL. Lastly, serum high-sensitivity C-reactive protein (hs-CRP) was measured using immunoturbidimetry with the Atellica® Solution CH, and serum interleukin 6 (IL-6), N-terminal pro-B-type natriuretic peptide (NT-proBNP), and high-sensitivity cardiac troponin T (hs-cTnT) were measured by electrochemiluminescence Elecsys® immunoassay method using a cobas® 6000 analyzer (Roche Diagnostics).

### Statistical analysis

From the initial 3273 participants, we excluded 483 with missing information or implausible energy intake (< 500 and > 4000 for women, and < 800 and > 5000 for men), 232 without GDF-15 measurements, and 61 with lacking data on potential confounders. Therefore, the final analytical sample included 2497 participants (Supplementary Figure S1). Baseline participants’ characteristics were summarized by quartiles of adherence to the PHDI, with the mean and standard deviation for continuous variables, and proportions for categorical ones.

The association between the PHDI and GDF-15 concentrations was assessed using multivariable linear regression models, where the dependent variable was the log-transformed GDF-15. The association was then summarized with mean percentage differences, calculated by subtracting 1 from the exponentiated β-coefficients resulting from the regression models and multiplying the result by 100, along with their 95% confidence interval (CI) across quartiles of adherence to the PHDI. Several consecutive models with incremental adjustment for confounders were fitted: Model 1 adjusted for sex, age, and educational level; Model 2 further adjusted for tobacco (current, past, never smoker), alcohol consumption (never, former, moderate and heavy drinker), energy intake, BMI, hours of TV, physical activity, diabetes, and CVD; Model 3 (main analysis model) further adjusted for systolic blood pressure, glucose, and LDL-C levels; and Model 4 further adjusted for log-transformed creatinine, NT-proBNP, hs-cTnT, and IL-6. To further understand if the association between PHDI and GDF-15 was somewhat biomarker-specific or simply reflected systemic inflammation, we also evaluated the association between quartiles of adherence to the PHDI and other inflammaging biomarkers such as IL-6 and hs-CRP, adjusting for the mentioned models with the addition of log-transformed GDF-15.

*P*-values for linear trend and dose–response associations between PHDI and GDF-15 concentrations were calculated using quartiles of PHDI as a continuous variable and evaluating the association per 20-point increase in PHDI’s adherence. Also, to assess the dose–response relationship and potential non-linearity, restricted cubic spline regression was used, adjusted as in Model 3.

In sensitivity analyses, the robustness of the results was tested as follows: (1) we first excluded participants with a diagnosis of CVD or diabetes at baseline, given that GDF-15 is a biomarker of chronic disease burden; (2) given the underlying inflammation associated with excess weight, we repeated the analyses excluding participants with obesity in addition to those with CVD or diabetes.

Additionally, we stratified the analyses by a few main covariates (sex, age groups, educational level, tobacco use, alcohol consumption, BMI, CVD, type 2 diabetes, TV hours, and tertiles of physical activity) and assessed potential interactions defined as the product of the PHDI by such variables.

Finally, to identify the main drivers of the association between PHDI and GDF-15 concentrations, we evaluated each item of the PHDI individually and assessed how excluding each item, one at a time, influenced the association.

Analyses were performed using Stata version 17.0 (Stata Corp LLC, College Station, TX). *p*-values were two-tailed and were considered statistically significant at *p* < 0.05.

## Results

Participants had a mean age (SD) of 71.6 years (4.4) and 52.9% were women. The mean (SD) score for the PHDI was 94.5 (9.8) for women and 92.5 (8.9) for men. Participants with the highest adherence to the PHDI were more likely to be women, less sedentary, with lower energy intake, BMI, SBP, glucose, and GDF-15 levels (Table 1). Compared to the analytical sample, excluded participants were more likely to be younger, with a lower PHDI score and physical activity, and a higher BMI, SBP, sedentary time, and frequency of chronic diseases (Supplementary Table S2).

**Table 1:** Baseline characteristics of older adults in the Seniors-ENRICA-2 study by quartiles of adherence to the PHDI

	Q1	Q2	Q3	Q4	Total
*n*	618	624	613	642	2497
*PHDI points*	81.8 (4.8)	90.3 (1.8)	96.3 (1.8)	105.4 (5.1)	93.6 (9.4)
Sex, women (%)	300 (48.5)	303 (48.6)	317 (51.7)	401 (62.5)	1321 (52.9)
Age, years	71.3 (4.5)	71.8 (4.4)	71.5 (4.3)	71.6 (4.4)	71.6 (4.4)
Education (%)					
Primary or less	377 (61.0)	406 (65.1)	404 (65.9)	398 (62.0)	1585 (63.5)
Secondary	125 (20.3)	112 (17.9)	109 (17.8)	123 (19.2)	469 (18.8)
University	116 (18.8)	106 (17.0)	100 (16.3)	121 (18.9)	443 (17.7)
Smoking status (%)					
Current	82 (13.3)	57 (9.1)	51 (8.3)	43 (6.7)	233 (9.3)
Former	245 (39.6)	230 (36.9)	236 (38.5)	238 (37.1)	949 (38.0)
Never	291 (47.1)	337 (54.0)	326 (53.2)	361 (56.2)	1315 (52.7)
Alcohol intake (%)					
Never	116 (18.8)	124 (19.9)	114 (18.6)	121 (18.9)	475 (19.0)
Former	30 (4.9)	41 (6.6)	42 (6.9)	45 (7.0)	158 (6.3)
Moderate	315 (51.0)	327 (52.4)	328 (53.5)	345 (53.7)	1315 (52.7)
Heavy	157 (25.4)	132 (21.2)	129 (21.0)	131 (20.4)	549 (22.0)
Energy intake, kcal/day	1987.2 (366.8)	1954.1 (348.2)	1934.9 (349.8)	1919.8 (338.9)	1948.7 (351.6)
BMI, kg/m^2^	28.1 (4.6)	28.0 (4.6)	27.6 (4.4)	27.3 (4.2)	27.8 (4.5)
TV hours	3.31 (1.6)	3.18 (1.5)	3.20 (1.6)	3.04 (1.5)	3.18 (1.6)
Physical activity*	26.5 (19.9)	27.7 (18.9)	28.8 (18.9)	29.2 (18.6)	28.1 (19.1)
T2D (%)	121 (19.6)	123 (19.7)	111 (18.1)	102 (15.9)	457 (18.3)
CVD (%)	18 (2.9)	20 (3.2)	25 (4.1)	21 (3.3)	84 (3.4)
SBP	134.6 (18.4)	134.7 (18.1)	135.2 (18.6)	133.7 (17.4)	134.5 (18.1)
Serumbiomarkers					
Glucose,mg/L	101.5 (26.3)	100.4 (24.1)	100.0 (23.9)	97.3 (19.5)	99.8 (23.6)
LDL-C, mg/dL	114.8 (28.6)	111.7 (28.0)	112.5 (29.6)	115.6 (29.6)	113.7 (29.0)
GDF-15,pg/mL	1513.4 (1194.8)	1518.3 (1109.1)	1442.0 (1036.9)	1314.3(827.9)	1445.9 (1051.9)

Continuous variables are expressed as mean (standard deviation). Categorical variables are expressed as percentages (%)

*PHDI* Planetary Health Diet Index, *Q* quartiles, *BMI* body mass index, *CVD* cardiovascular disease, *T2D* type 2 diabetes, *SBP* systolic blood pressure, *LDL-C* low-density lipoprotein cholesterol, *GDF-15* growth differentiation factor-15

^*^Expressed as metabolic equivalents of task-hour/week

**Fig. 1:**
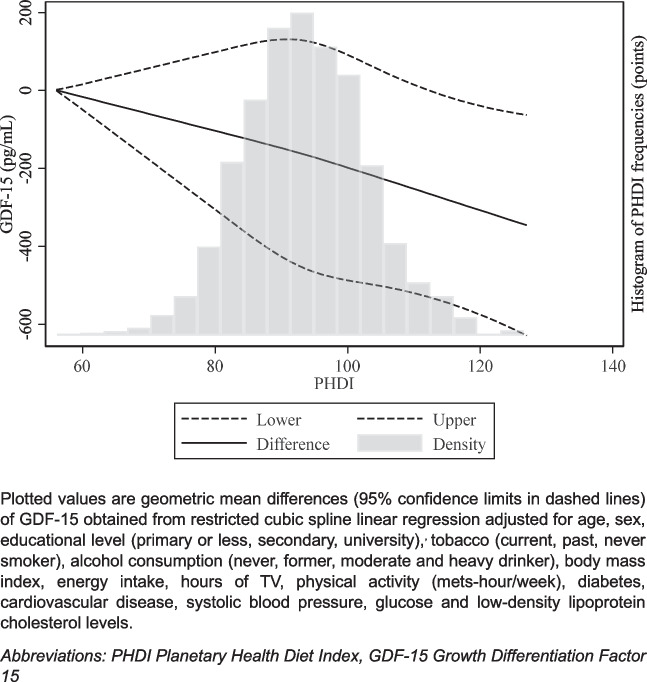
Dose–response association of the PHDI with GDF-15 concentrations (pg/mL). *N* = *2497*

Higher adherence to the PHDI was associated with lower levels of GDF-15, in a dose–response manner (Table 2). When comparing the fourth vs the first quartile of adherence to the PHDI, the mean percentage difference (95% CI) in GDF-15 in the main analysis model was − 6.8% (− 11.1, − 2.4). The mean percentage difference (95% CI) per 20-point increase of adherence was − 4.4% (− 7.7, − 0.9) potentially suggesting that increasing adherence to just 2 of the PHDI recommendations could already lead to significant reductions in GDF-15 levels. Moreover, in the restricted cubic spline regression analysis, PHDI and GDF-15 showed an inverse linear dose–response relationship (Fig. 1).

**Table 2:** Mean percentage differences (95% confidence interval) in GDF-15 concentrations by quartiles of adherence to the PHDI. *N* = *2497*

	Q1 (lowest)	Q2	Q3	Q4 (highest)	*p*-trend	Per 20-point increase
*N*	618	624	613	642		
PHDI score	81.8 (4.8)	90.3 (1.8)	96.3 (1.8)	105.4 (5.1)		
Model 1^a^	1 Ref	− 1.7 (− 6.7,3.6)	− 3.8 (− 8.7,1.4)	− 9.4 (− 14.0, − 4.6)	*p* < 0.001	− 6.7 (− 10.3, − 3.0)
Model 2^b^	1 Ref	− 0.8 (− 5.4,4.0)	− 2.2 (− 6.8,2.5)	− 6.7 (− 11.0, − 2.2)	*p* < 0.001	− 4.3 (− 7.7, − 0.8)
Model 3^c^	1 Ref	− 1.4 (− 5.9,3.4)	− 2.9 (− 7.3,1.8)	− 6.8 (− 11.1, − 2.4)	*p* < 0.001	− 4.4 (− 7.7, − 0.9)
Model 4^d^	1 Ref	− 1.8 (− 5.9,2.6)	− 3.2 (− 7.4,1.1)	− 6.0 (− 10.0, − 1.9)	*p* < 0.001	− 4.5 (− 7.6, − 1.3)

Values of all models are geometric means (95% CI)

*PHDI* Planetary Health Diet Index*, Q* quartiles*, CI* confidence interval, *GDF-15* growth differentiation factor-15

^a^Age, sex, and educational level (primary or less, secondary, university)

^b^Model 1 further adjusted for tobacco (current, past, never smoker), alcohol consumption (never, former, moderate and heavy drinker), body mass index, energy intake, hours of TV, physical activity (mets-hour/week), diabetes, and cardiovascular disease

^c^Model 2 further adjusted for systolic blood pressure, glucose, and LDL-C

^d^Model 3 further adjusted for log-transformed creatinine, NT-proBNP, hs-cTnT, and IL-6

Of note, the association between the PHDI and GDF-15 was independent of other related biomarkers (creatinine, NT-proBNP, hs-cTnT, and IL-6), as shown in Model 4 (Table 2). In addition, the mean percentage difference (95% CI) between the PHDI (Q1 vs Q4) and other inflammation biomarkers was − 10.8% (− 16.3, − 5.1) for IL-6, and − 30.7% (− 44.9, − 12.7) for hs-CRP (Supplementary Table S3), which indicates that the association between the PHDI and these biomarkers is stronger than that found for the PHDI and GDF-15. In sensitivity analyses, the association between the PHDI and GDF-15 remained in the same direction: (1) when we excluded participants with CVD or diabetes at baseline (Supplementary Table S4), (2) when further excluding participants with obesity; indeed, the mean percentage difference (95% CI) was increased to − 8.1% (− 13.2, − 2.7) (Supplementary Table S5). Overall, these results suggest that adherence to the PHDI is associated with lower GDF-15 concentrations—independently of other related biomarkers, health, and weight status—and may also reduce additional inflammatory markers, highlighting the beneficial impact of this dietary pattern on inflammaging-related biomarkers.

In stratified analyses, participants that were older (≥ 75 years), men, non-obese, with a lower educational level or less sedentary, and drinkers (moderate and heavy) seemed to benefit most from a higher adherence to the PHDI, as they showed a larger mean percentage decrease of GDF-15 serum concentrations. No significant interactions were found but for BMI (*p* = 0.030), where the association seemed to be stronger for individuals with a healthy weight (Supplementary Table S6).

**Fig. 2:**
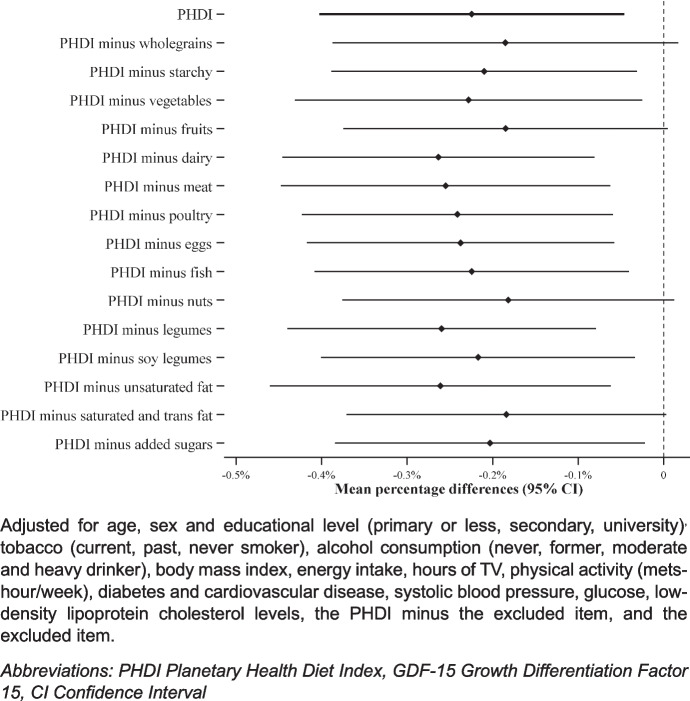
Association of the PHDI and the PHDI excluding each food group at a time, with GDF-15 concentrations. Results are expressed in mean percentage difference (95% confidence interval) in GDF-15 concentrations

Lastly, regarding the analysis of each individual component of the PHDI, we observed that excluding the items concerning PHDI’s recommendations for whole grains, fruits, nuts, and trans or saturated fat, one at a time, resulted in a loss of the statistical significance of the association between the PHDI and GDF-15 (Fig. 2), which may indicate that these food groups are key to the observed benefits.

## Discussion

In this cohort of Spanish older adults, higher adherence to the PHDI was associated with lower serum concentrations of GDF-15. This association was independent of risk factors and biomarkers of chronic disease and remained robust for other related biomarkers, such as IL-6 and hs-CRP, and when excluding participants with CVD, diabetes, and obesity. Overall, our results suggest that even older adults free of chronic diseases can benefit from this plant-based and environmentally friendly dietary pattern.

While the PHDI has been designed to tackle our health and environmental challenges [10], knowledge on its effects on health outcomes and inflammatory-driven diseases remains unexplored in many populations, especially in older individuals, who have a higher risk for chronic diseases partly because of age-associated inflammation biomarkers [31].

Previous observational studies have found that, overall, adherence to plant-based diets (PBD), such as the DASH [7] and Mediterranean diet [7] result in lower GDF-15 levels, which are comparable to ours for PHDI (mean percentage differences [95% CI] − 4.4 [− 8.5, − 0.0] and − 4.9 [− 8.9, − 0.8], respectively). In this context, the PHDI has been associated with a lower risk of CVD [13, 14], cancer [32, 33], diabetes [14, 15], and overall and cause-specific mortality [16–18, 34] as well as a reduced environmental burden [16, 35] across several populations. Given the increased risk of these conditions that come along with elevated levels of GDF-15 and other inflammatory biomarkers [3, 4, 19–21], their modulation through adherence to the PHDI, as observed in this study, may play a useful role in terms of NCD prevention and planetary health. In the Malmö Diet and Cancer cohort of middle-aged adults [9], adherence to the PHDI was inversely associated with GDF-15 (*β* coefficients; 95% CI − 0.25; − 0.36, − 0.13) and IL-6 (− 0.21; − 0.31, − 0.10) concentrations, which were in turn associated with a higher risk of heart failure. These findings go in line with our results, reflecting that, in middle-aged healthy populations, adherence to the PHDI reduces the concentrations of GDF-15 and related biomarkers. Nevertheless, our findings extend further, revealing these benefits in older populations. Moreover, to estimate adherence to the EAT-Lancet proposed dietary pattern [10], whereas authors in the Swedish cohort used a score (0–42 points) categorized in 4 levels of adherence [17], we used a continuous and prorated score [16] (0–140 points), which may better capture intermediate intake levels and, as such, reflect the actual dietary intake of the study population.

The associations in our study remained robust even when adjusting for other related biomarkers, suggesting that the association between the PHDI and GDF-15 is partly due to mechanisms specific to the GDF-15 and not only related to systemic inflammation or cardiac-renal disease burden per se. In this line, IL-6 and hs-CRP concentrations were also inversely and significantly associated with adherence to the PHDI. Both IL-6 and hs-CRP are key inflammatory biomarkers that have been shown to predict CVD and all-cause death risk [36–38]. These results are consistent with previous research suggesting that PBD may favorably influence biomarkers related to inflammaging [7, 39–41].

In addition, we observed a stronger association in certain population subgroups, such as older or healthy individuals, as well as those with an optimal weight. These findings might be partially explained by metabolic or lifestyle-related factors, such as sex-specific inflammatory responses, age-related increases in GDF-15, or greater metabolic flexibility in non-obese individuals, which may modulate the impact of diet on this biomarker. Moreover, individuals with obesity often have a chronic low-grade inflammatory state that may mask the anti-inflammatory benefits of a healthy diet, such as the PHDI. This aligns with previous research suggesting that dietary changes alone (without weight loss or other lifestyle interventions) may not be sufficient to reverse inflammation in individuals with excess adiposity [39]. Similarly, our results remained in healthy individuals without CVD or diabetes, underscoring the potential of the PHDI to help prevent chronic diseases. Although no interaction was found for age, participants over 75 seemed to benefit most from adhering to the PHDI. This is of high relevance, especially in countries like Spain, where life expectancy continues to increase [42].

Although the benefits of the PHDI on GDF-15 levels may primarily result from the combined effect of all its encompassing components, we found that following the recommendations for whole grains, fruits, nuts, or trans and saturated fats were the main driver of the observed association. Fruits, whole grains, and nuts are naturally high in fiber and are good sources of fat, minerals, vitamins, and other bioactive compounds with antioxidant and anti-inflammatory properties [10]. Overall, increased dietary fiber intake has been linked to lower levels of inflammatory markers, such as IL-6 [43], TNF-α [43], and hs-CRP [44]. Moreover, the substitution of staple foods with whole grains has been associated with lower pro-inflammatory biomarkers [45], as has every 5-g increase in daily cereal fiber intake on hs-CRP concentrations [46]. As regards nuts, they are high in fiber and also in unsaturated fatty acids that have been associated with reduced oxidative stress [47] and inflammation [47]. Indeed, in the Spanish PREDIMED trial [48], participants randomly assigned to 30 g/day intake of nuts had a 28% lower CVD risk, whose etiology is rooted in inflammation [49] where GDF-15 is highly expressed [3]. Thus, the recommended intake of nuts in PHDI (≥ 50 g/day, or a handful of nuts) may account for its key role in its association with GDF-15. In addition, trans fats have been associated with an increased risk of CVD [50], and saturated fats have been positively linked to inflammation [51] and activation of GDF-15 [52], which may thereby explain why reducing the intake of hydrogenated vegetable oils, butter, or margarine may also be important in the association between the PHDI and lower GDF-15. Lastly, previous literature has shown that the replacement of animal protein with vegetable protein may be beneficial in older adults and could thus represent another mechanism through which the PHDI may improve GDF-15 levels [53, 54]. Overall, while whole grains, fruits, nuts, and low intake of trans and saturated fats emerged as relevant contributors, the association between PHDI and GDF-15 weakened when each component was removed from the overall score, suggesting that no single component solely drives the association. This supports the idea of a synergistic effect among dietary components, which is consistent with the multidimensional nature of healthy dietary patterns.

The strengths of this study include the use of validated assessment tools, such as the ENRICA dietary history [26] and the EPIC physical activity questionnaire [29]. Also, given the wide variety of indexes that measure adherence to the PHD in the literature, the PHDI score used in this study, as defined by Bui et al. [16], is likely to better capture the variability in dietary intake, given its large range (0 to 140 points) and its ability to prorate.

Several limitations should also be acknowledged. First, given the cross-sectional design of the study, the temporal relation between GDF-15 and PHDI could not be established, limiting the ability to draw causal inferences. However, our results align with previous literature, in which GDF-15 serum concentrations have been inversely associated with adherence to other PBDs [7] and positively linked with CVD [1, 2, 9, 20] and mortality [55]. Second, data on some variables such as dietary intake were self-reported, so there might be some degree of misclassification, although probably non-differential [26]. Third, as in any observational study, some residual confounding may persist despite the fact that we adjusted for a good number of potential confounders, including risk factors and inflammation biomarkers of chronic diseases. Lastly, this study was conducted among predominantly white older adults from a Mediterranean country, which may limit the generalizability of our findings to more ethnically diverse populations.

## Conclusion

In conclusion, higher adherence to the PHDI was associated with lower serum concentrations of GDF-15 in older adults. Given the growing aging population, the PHDI, a flexible PBD that can be adopted by different populations across the globe and in different cultural contexts, is a promising strategy for lowering chronic inflammation and, subsequently, reducing NCD, while staying within planetary boundaries.

## Supplementary Information

Below is the link to the electronic supplementary material.

Supplementary file1 (PDF 371 KB)

Supplementary file2 (DOCX 62 KB)

## Data Availability

Data described in the manuscript, code book, and analytic code will be made available upon reasonable request.
