# Supplementary material for: Association between the Planetary Health Diet Index and growth differentiation factor-15: the Seniors ENRICA-2 cohort
Source: GeroScience. 2025 May 31;48(1):679–90. doi: 10.1007/s11357-025-01712-8 (PMC12972292; doi:10.1007/s11357-025-01712-8)
Supplement: Supplementary file 1 — Supplementary file1 (PDF 371 KB) [file 11357_2025_1712_MOESM1_ESM.pdf]

Supplementary Figure S1. Flow diagram of included participants from the Seniors ENRICA-2 cohort

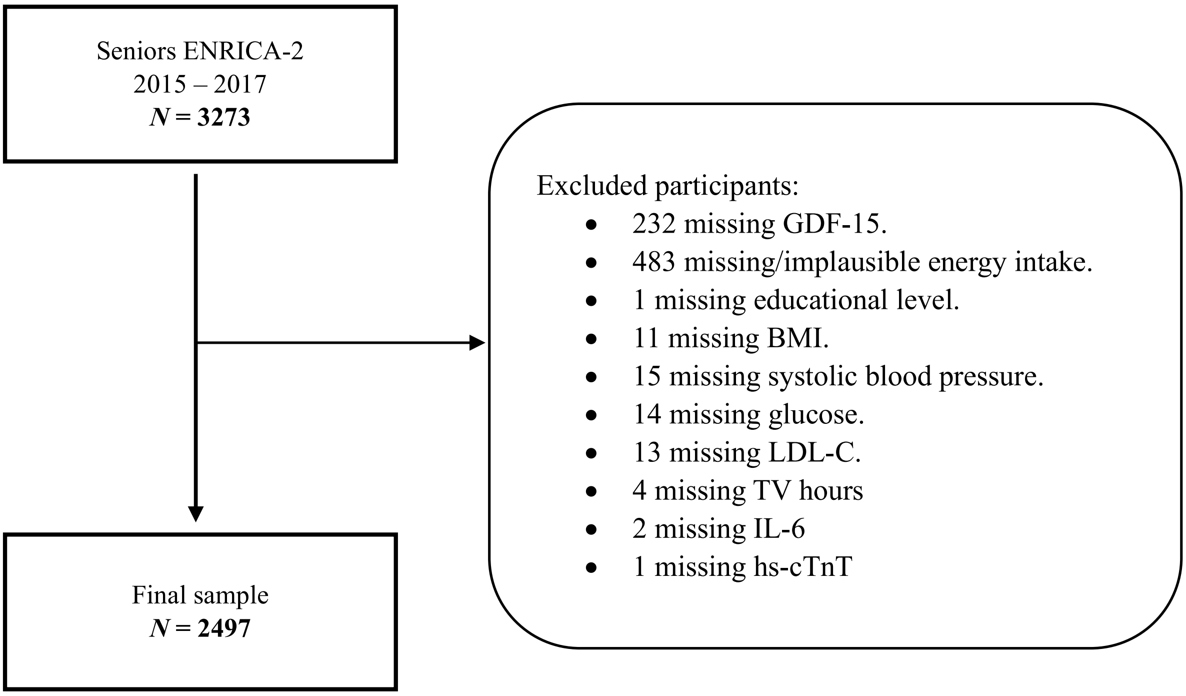

Abbreviations: BMI body mass index, GDF-15 Growth Differentiation factor 15, hs-cTnT high-sensitivity cardiac troponin T, IL-6 interleukin 6, LDL-C low-density lipoprotein cholesterol,.
