# Supplementary material for: Association between the Planetary Health Diet Index and growth differentiation factor-15: the Seniors ENRICA-2 cohort
Source: GeroScience. 2025 May 31;48(1):679–90. doi: 10.1007/s11357-025-01712-8 (PMC12972292; doi:10.1007/s11357-025-01712-8)
Supplement: Supplementary file 2 — Supplementary file2 (DOCX 62 KB) [file 11357_2025_1712_MOESM2_ESM.docx]

**TITLE: Association between the Planetary Health Diet Index and growth differentiation factor 15: The seniors ENRICA-2 cohort**

**Authors:** María del Carmen Aznar de la Riera, MSc^1^, Rosario Ortolá^1,2^, MD, PhD, Blanca Fabre-Estremera, MSc^1,3,4^, Antonio Buño-Soto, MD, PhD^3,4^, Fernando Rodríguez-Artalejo^1,2,5^ MD, PhD, Mercedes Sotos-Prieto^1,2,5,6^, PhD

^1^ Department of Preventive Medicine and Public Health. School of Medicine. Universidad Autónoma de Madrid, Avda del Arzobispo Morcillo, 4. 28029, Madrid, Spain.

^2^ CIBERESP (CIBER of Epidemiology and Public Health), Av. Monforte de Lemos, 3-5. 28029, Madrid, Spain.

^3^ Department of Laboratory Medicine, La Paz University Hospital, Madrid, 28046, Spain

^4^ IdiPaz-Hospital La Paz Institute for Health Research, Madrid, Spain.

^5^ IMDEA-Food Institute. CEI UAM+CSIC, Ctra. de Canto Blanco 8, E. 28049, Madrid, Spain.

^6^ Department of Environmental Health, Harvard T.H. Chan School of Public Health, 665 Huntington Avenue. Boston, Massachusetts 02115, USA.

**Corresponding author:** Mercedes Sotos-Prieto, PhD

Department of Preventive Medicine and Public Health, School of Medicine, Universidad Autónoma de Madrid, Calle del Arzobispo Morcillo 4, 28029 Madrid, Spain. Telephone: (+34) 91 497 54 41. E-mail: [mercedes.sotos@uam.es](mailto:mercedes.sotos@uam.es)

**SUPPLEMENTARY TABLES AND FIGURES:**

**Supplementary Table S1.** Description of the Planetary Health Diet Index (PHDI)…………………………………….2

**Supplementary Table S2.** Differences in participant’s characteristics by inclusion status in the Seniors-ENRICA-2 cohort. ……………………………………………………………………………………………………………………3

**Supplementary Table S3.** Mean percentage differences (95% confidence interval) in some inflammaging biomarkers concentrations by quartiles of adherence to the PHDI…………………………………………………………………...4

**Supplementary Table S4.** Mean percentage differences (95% confidence interval) in GDF-15 concentrations by quartiles of adherence to the PHDI excluding participants **with CVD or diabetes** at baseline. *N= 1979…………………5*

**Supplementary Table S5.** Mean percentage differences (95% confidence interval) in GDF-15 concentrations by quartiles of adherence to the PHDI excluding participants with **diabetes, CVD or BMI≥30**. *N= 1512………………………………………………………………………………………………………...…………………………...6*

**Supplementary Table S6.** Subgroups mean percentage differences (95% confidence interval) in GDF-15 concentrations by quartiles of adherence to the PHDI. *N= 2497…..…………………………………………………………...7*

**Supplementary Figure S1.** Flow diagram of included participants from the Seniors ENRICA-2 cohort……………….9

**Supplementary Table S1.** Description of the Planetary Health Diet Index (PHDI)

| Nº | Component | EAT-Lancet reference diet  (for 2500 kcal/d demand) | | Criteria for scoring for PHDI (range 0-140) | | |
| --- | --- | --- | --- | --- | --- | --- |
|  |  | gram/day (uncertainty range) | kcal/day | Min score (0) in gram/d | Max score (10) in gram/d | Weight in total score |
| 1 | Whole grain (dry weight) | 232 (0-60% of TEI) | 811 | 0 | ≥ 75 for women ≥ 90 for men | 1 |
| 2 | Starchy vegetables such as potatoes, cassava | 50 (0-100) | 39 | ≥ 200 | ≤ 50 | 1 |
| 3 | Vegetable (not including potato or other starchy vegetables) | 300 (200-600) | 78 | 0 | ≥ 300 | 1 |
| 4 | Whole fruit (not including fruit juice) | 200 (100-300) | 126 | 0 | ≥ 200 | 1 |
| 5 | Dairy foods (e.g., milk, cheese) | 250 (0-500) | 153 | ≥ 1000 | ≤ 250 | 1 |
| 6 | Red/processed meat (e.g., beef, lamb, pork) | 14 (0-28) | 30 | ≥ 100 | ≤ 14 | 1 |
| 7 | Chicken and other poultry (e.g., turkey, duck, goose, ostrich) | 29 (0-58) | 62 | ≥ 100 | ≤ 29 | 1 |
| 8 | Eggs (with shell) | 13 (0-25) | 19 | ≥ 120 | ≤ 13 | 1 |
| 9 | Fish/shellfish | 28 (0-100) | 40 | 0 | ≥ 28 | 1 |
| 10 | Nuts (peanuts, tree nuts- - e.g., walnut, almond, hazelnuts, pecan, cashew, pistachio) | 50 (0-75) | 291 | 0 | ≥ 50 | 1 |
| 11 | Non soy legumes (e.g., dry beans, lentils, peas) | 50 (0-100) | 172 | 0 | ≥100 | 0.5 |
| 12 | Soybean/ soy foods (dry weight) | 25 (0-50) | 112 | 0 | ≥50 | 0.5 |
| 13 | Added fat – Unsaturated oils (e.g., olive soybean, rapeseed, sunflower, peanut oil) | 40 (20-80) | 354 (14.16% of TEI) | ≤ 3.5% of TEI | ≥ 21% of TEI | 1 |
| 14 | Added fat – Saturated oils, trans fat (e.g., palm oil, coconut oil, dairy fat (butter), margarine, lard, tallow) | 11.8 (0-11.8) | 96 (3.8% of TEI) | ≥10% of TEI | 0% of TEI | 1 |
| 15 | Added sugar and fruit juice | 31 (0-31) | 120 (4.8% of TEI) | ≥25% of TEI | ≤ 5% of TEI | 1 |

*Abbreviations: PHDI Planetary Health Diet Index***,** TEI Total Energy Intake

**Supplementary Table S2.** Differences in participant’s characteristics by inclusion status in the Seniors-ENRICA-2 cohort.

|  | Included participants | Excluded participants due to missing GDF-15 data |
| --- | --- | --- |
| *n* (%) | 2497 | 232 |
| PHDI points | 93.6 (9.4) | 92.1 (8.8) |
| Sex, women, (%) | 1321 (52.9) | **106 (45.7)** |
| Age, years | 71.6 (4.4) | **73.2 (4.6)** |
| Education, (%) |  |  |
| Primary or less | 1585 (63.5) | 156 (67.5) |
| Secondary | 469 (18.8) | 34 (14.7) |
| University | 443 (17.7) | 41 (17.8) |
| Smoking Status, (%) |  |  |
| Current | 233 (9.3) | 20 (8.6) |
| Former | 949 (38.0) | 105 (45.3) |
| Never | 1315 (52.7) | 107 (46.1) |
| Alcohol Intake, (%) |  |  |
| Never | 475 (19.0) | 36 (15.5) |
| Former | 158 (6.3) | 20 (8.6) |
| Moderate | 1315 (52.7) | 135 (58.2) |
| Energy intake, kcal/day | 1948.7 (351.6) | 1978.6 (365.4) |
| BMI, kg/m^2^ | 27.8 (4.5) | **29.5 (5.0)** |
| TV hours | 3.2 (1.6) | **3.5 (1.7)** |
| Physical activity* | 28.1 (19.1) | **24.7 (18.6)** |
| T2D, (%) | 457 (18.3) | 49 (21.1) |
| CVD, (%) | 84 (3.4) | **43 (18.5)** |
| SBP | 134.5 (18.1) | **131.7 (20.9)** |
| Serum biomarkers |  |  |
| Glucose, mg/dL | 99.8 (23.6) | 104.6 (35.8) |
| LDL-C, mg/dL | 113.7 (29.0) | 109.1 (41.7) |

*Continuous variables are expressed as mean (standard deviation) and categorical variables as percentages (%).*

*Abbreviations: PHDI Planetary Health Diet Index, GDF-15 Growth Differentiation Factor 15, BMI body mass index, CVD cardiovascular disease, T2D type 2 diabetes, SBP systolic blood pressure, LDL-C low density lipoprotein cholesterol*

**Expressed as metabolic equivalents of task-hour/week*

**Supplementary Table S3.** Mean percentage differences (95% confidence interval) in some inflammaging biomarkers concentrations by quartiles of adherence to the PHDI.

|  | Q1 (lowest) | Q2 | Q3 | Q4 (highest) | p-trend | Per 20-point increase |
| --- | --- | --- | --- | --- | --- | --- |
| **IL-6** |  |  |  |  |  |  |
| N | 618 | 624 | 613 | 642 |  |  |
| PHDI score | 81.8 (4.8) | 90.3 (1.8) | 96.3 (1.8) | 105.4 (5.1) |  |  |
| Model 1 ^a^ | 1 Ref. | -3.3(-9.3,3.1) | **-11.0(-16.5,-5.1)** | **-13.7(-19.0,-8.0)** | **p<0.001** | **-9.6 (-13.8,-5.1)** |
| Model 2 ^b^ | 1 Ref. | -2.3(-8.2,4.1) | **-9.2(-14.7,-3.2)** | **-10.9(-16.4,-5.1)** | **p<0.001** | **-6.7(-11.1,-2.2)** |
| Model 3 ^c^ | 1 Ref. | -2.5(-8.4,3.8) | **-9.5(-15.0,-3.6)** | **-10.8(-16.3,-5.1)** | **p<0.001** | **-6.7 (-11.0,-2.1)** |
| Model 4 ^d^ | 1 Ref. | -2.2(-8.0,4.0) | **-9.5(-14.9,-3.7)** | **-9.8(-15.2,-4.0)** | **p<0.001** | **-6.3 (-10.6,-1.8)** |
| **hs-CRP** |  |  |  |  |  |  |
| N | 320 | 280 | 257 | 248 |  |  |
| PHDI score | 81.4 (5.2) | 90.3 (1.8) | 96.3 (1.8) | 104.9 (4.9) |  |  |
| Model 1 ^a^ | 1 Ref. | -18.9(-35.2,1.5) | **-25.0(-40.4,-5.6)** | **-37.1(-50.2,-20.6)** | **p<0.001** | **-30.9(-42.0,-17.6)** |
| Model 2 ^b^ | 1 Ref. | -13.0(-30.3,8.6) | **-21.5(-37.4,-1.5)** | **-30.3(-44.7,-12.2)** | **p<0.001** | **-24.7(-36.8,-10.3)** |
| Model 3 ^c^ | 1 Ref. | -12.5(-29.8,9.1) | **-21.1(-37.0,-1.1)** | **-30.7(-44.9,-12.7)** | **p<0.001** | **-25.1(-37.1,-10.9)** |
| Model 4 ^e^ | 1 Ref. | -8.1(-24.9,12.4) | -15.3(-31.1,4.1) | **-24.9(-39.2,-7.2)** | **p<0.001** | **-23.0(-34.4,-9.7)** |

*Values of all models are geometric means (95% CI)*

*Abbreviations: PHDI: Planetary Health Diet Index, Q quartile, CI confidence Interval, GDF-15 Growth Differentiation Factor 15, IL-6: Interleukin 6, hs-CRP: High Sensitivity C-Reactive Protein*

^a^ Age, sex and educational level (primary or less, secondary, university)

^b^ Model 1 further adjusted for tobacco (current, past, never smoker), alcohol consumption (never, former, moderate and heavy drinker), body mass index, energy intake, hours of TV, physical activity (mets-hour/week), diabetes and cardiovascular disease.

^c^ Model 2 further adjusted for systolic blood pressure, glucose and LDL-C levels.

^d^ Model 3 further adjusted for log-transformed creatinine, NT-proBNP, hs-cTnT, and GDF-15 concentrations.

^e^ Model 3 further adjusted for log-transformed creatinine, NT-proBNP, hs-cTnT, GDF-15 and IL-6 concentrations.

**Supplementary Table S4.** Mean percentage differences (95% confidence interval) in GDF-15 concentrations by quartiles of adherence to the PHDI excluding participants **with CVD or diabetes** at baseline. *N= 1979*

|  | Q1 (lowest) | Q2 | Q3 | Q4 (highest) | p-trend | Per 20-point increase |
| --- | --- | --- | --- | --- | --- | --- |
| N | 483 | 486 | 483 | 527 |  |  |
| PHDI score | 81.7 (4.7) | 90.3 (1.8) | 96.3 (1.8) | 105.4 (5.2) |  |  |
| Model 1 ^a^ | 1 Ref. | -1.0 (-5.8,4.0) | -3.2 (-7.9,1.8) | **-7.6 (-12.0,-2.9)** | **p<0.001** | **-4.6 (-8.0,-1.0)** |
| Model 2 ^b^ | 1 Ref. | 0.1 (-4.7,5.2) | -1.7 (-6.4,3.3) | **-6.1 (-10.5,-1.4)** | **p<0.001** | -3.1 (-6.6,0.5) |
| Model 3 ^c^ | 1 Ref. | -0.1 (-4.9,4.9) | -2.0 (-6.7,3.0) | **-5.9 (-10.4,-1.3)** | **p<0.001** | -3.0 (-6.5,0.6) |
| Model 4 ^d^ | 1 Ref. | -1.2 (-5.5,3.2) | -1.9 (-6.2,2.5) | **-5.6 (-9.7,-1.4)** | **p<0.001** | **-3.3 (-6.5,-0.0)** |

*Values of all models are geometric means (95% CI)*

*Abbreviations: PHDI Planetary Health Diet Index, Q quartiles, CI Confidence Interval, GDF-15 Growth Differentiation Factor 15, CVD cardiovascular disease*

^a^ Age, sex and educational level (primary or less, secondary, university)

^b^ Model 1 further adjusted for tobacco (current, past, never smoker), alcohol consumption (never, former, moderate and heavy drinker), body mass index, energy intake, hours of TV, physical activity (mets-hour/week), diabetes and cardiovascular disease.

^c^ Model 2 further adjusted for systolic blood pressure, glucose and LDL-C levels.

^d^ Model 3 further adjusted for log-transformed creatinine, NT-proBNP, hs-cTnT, and IL-6 concentrations.

**Supplementary Table S5.** Mean percentage differences (95% confidence interval) in GDF-15 concentrations by quartiles of adherence to the PHDI excluding participants with **diabetes, CVD or BMI≥30**. *N= 1512*

|  | Q1 (lowest) | Q2 | Q3 | Q4 (highest) | p-trend | Per 20-point increase |
| --- | --- | --- | --- | --- | --- | --- |
| N | 360 | 370 | 375 | 407 |  |  |
| PHDI score | 81.8 (4.6) | 90.3 (1.8) | 96.4 (1.8) | 105.6 (5.4) |  |  |
| Model 1 ^a^ | 1 Ref. | **-6.2 (-11.5,-0.6)** | -5.4 (-10.7,0.3) | **-9.8 (-14.9,-4.5)** | **p<0.001** | **-5.7 (-9.6,-1.5)** |
| Model 2 ^b^ | 1 Ref. | -4.8 (-10.1,0.8) | -4.0 (-9.4,1.7) | **-8.5 (-13.6,-3.2)** | **p<0.001** | **-4.5 (-8.5,-0.3)** |
| Model 3 ^c^ | 1 Ref. | -5.0 (-10.2,0.6) | -4.2 (-9.5,1.5) | **-8.1 (-13.2,-2.7)** | **p<0.001** | -4.1 (-8.1,0.0) |
| Model 4 ^d^ | 1 Ref. | -4.9 (-9.7,0.2) | -4.3 (-9.1,0.9) | **-8.1(-12.7,-3.2)** | **p<0.001** | **-4.7 (-8.3,-0.9)** |

*Values of all models are geometric means (95% CI)*

*Abbreviations: PHDI: Planetary Health Diet Index, Q quartiles, CI confidence Interval, GDF-15 Growth Differentiation Factor 15, CVD cardiovascular disease, BMI body mass index*

^a^ Age, sex and educational level (primary or less, secondary, university)

^b^ Model 1 further adjusted for tobacco (current, past, never smoker), alcohol consumption (never, former, moderate and heavy drinker), body mass index, energy intake, hours of TV, physical activity (mets-hour/week), diabetes and cardiovascular disease.

^c^ Model 2 further adjusted for systolic blood pressure, glucose and LDL-C levels.

^d^ Model 3 further adjusted for log-transformed creatinine, NT-proBNP, hs-cTnT, and IL-6 concentrations.

**Supplementary Table S6.** Subgroups mean percentage differences (95% confidence interval) in GDF-15 concentrations by quartiles of adherence to the PHDI. *N= 2497*

|  | *N (%)* | **Q1** (lowest) | **Q2** | **Q3** | **Q4** (highest) | P for interaction |
| --- | --- | --- | --- | --- | --- | --- |
| Sex |  |  |  |  |  | 0.707 |
| Men | 1176 (47.1) | 1 Ref. | -1.8 (-8.1,5.0) | -0.3 (-6.9,6.8) | **-9.7 (-16.0,-2.9)** |  |
| Women | 1321 (52.9) | 1 Ref. | -1.3 (-7.6,5.4) | -6.0 (-12.0,0.3) | -5.1 (-10.8,1.0) |  |
| Age |  |  |  |  |  | 0.523 |
| <75 | 1885 (75.5) | 1 Ref. | -0.6 (-5.8,4.9) | -1.7 (-6.8,3.7) | **-5.5 (-10.4,-0.3)** |  |
| ≥75 | 612 (24.5) | 1 Ref. | -3.1 (-12.2,7.0) | -6.7 (-15.5,3.2) | **-10.6 (-19.2,-1.2)** |  |
| Education |  |  |  |  |  | 0.762 |
| Primary | 1585 (63.5) | 1 Ref. | **-6.5 (-11.7,-1.0)** | **-6.3 (-11.5,-0.7)** | **-8.8 (-14.0,-3.3)** |  |
| Secondary | 469 (18.8) | 1 Ref. | 10.0 (-2.2,23.7) | 3.0 (-8.4,15.9) | -1.5 (-12.1,10.5) |  |
| University | 443 (17.7) | 1 Ref. | 4.3 (-7.2,17.2) | 1.5 (-9.8,14.3) | -4.9 (-15.1,6.5) |  |
| Tobacco |  |  |  |  |  | 0.550 |
| Current | 233 (9.3) | 1 Ref. | 4.8 (-10.9,23.3) | -8.7 (-22.9,8.2) | -12.3 (-26.7,5.0) |  |
| Fromer | 949 (38.0) | 1 Ref. | -1.5 (-8.7,6.3) | 1.0 (-6.3,9.0) | -5.4 (-12.3,2.1) |  |
| Never | 1315 (52.7) | 1 Ref. | -1.2 (-7.4,5.4) | -4.4 (-10.4,2.1) | **-6.2 (-12.1,-0.1)** |  |
| Alcohol |  |  |  |  |  | 0.721 |
| Never drinker | 475 (19.0) | 1 Ref. | **13.9 (1.4,27.8)** | -2.3 (-13.2,10.0) | -0.9 (-11.9,11.4) |  |
| Former drinker | 158 (6.3) | 1 Ref. | 14.5 (-7.7,41.9) | 7.8 (-13.0,33.5) | -1.0 (-19.8,22.2) |  |
| Moderate drinker | 1315 (52.7) | 1 Ref. | **-6.4 (-12.2,-0.3)** | **-6.3 (-12.1,-0.2)** | **-7.3 (-13.0,-1.2)** |  |
| Heavy Drinker | 549 (22.0) | 1 Ref. | -4.3 (-13.0,5.3) | 4.1 (-5.3,14.6) | **-11.4 (-19.5,-2.5)** |  |
| BMI |  |  |  |  |  | **0.030** |
| <25 | 670 (26.8) | 1 Ref. | -5.4 (-14.6,4.9) | **-9.5 (-18.1,-0.1)** | **-11.4 (-19.7,-2.4)** |  |
| 25-29.9 | 1175 (47.1) | 1 Ref. | -5.5 (-11.4,0.9) | -2.7 (-8.9,4.0) | **-8.4 (-14.3,-2.1)** |  |
| ≥30 | 652 (26.1) | 1 Ref. | 8.5 (-0.7,18.5) | 2.2 (-6.6,11.8) | -0.6 (-9.3,8.9) |  |
| CVD |  |  |  |  |  | 0.425 |
| Yes | 84 (3.4) | 1 Ref. | -25.0 (-45.8,3.8) | -15.1 (-37.7,15.6) | -14.4 (-39.0,20.2) |  |
| No | 2413 (96.6) | 1 Ref. | -0.2 (-4.8,4.7) | -2.5 (-7.1,2.3) | **-6.4 (-10.7,-1.8)** |  |
| Diabetes |  |  |  |  |  | 0.994 |
| Yes | 457 (18.3) | 1 Ref. | -3.6 (-15.6,10.3) | -7.9 (-19.7,5.6) | -9.8 (-21.7,3.8) |  |
| No | 2040 (81.7) | 1 Ref. | -1.4 (-6.0,3.5) | -2.2 (-6.8,2.7) | **-6.5 (-10.9,-1.9)** |  |
| TV hours |  |  |  |  |  | 0.092 |
| ≤2 hours | 805 (32.2) | 1 Ref. | -5.2 (-12.9,3.2) | -7.7 (-15.0,0.3) | **-10.3 (-17.4,-2.6)** |  |
| >2 hours | 1692 (67.7) | 1 Ref. | 0.2 (-5.4,6.0) | -0.6 (-6.2,5.3) | -5.4 (-10.7,0.2) |  |
| Physical Activity* |  |  |  |  |  | 0.709 |
| Tertile 1 | 962 (38.5) | 1 Ref. | 0.4 (-7.1,8.6) | 0.2 (-7.6,8.6) | -3.4 (-11.0,4.7) |  |
| Tertile 2 | 717 (28.7) | 1 Ref. | 2.0 (-6.7,11.6) | -2.3 (-10.5,6.7) | **-10.0 (-17.5,-1.7)** |  |
| Tertile 3 | 818 (32.8) | 1 Ref. | -6.3 (-13.4,1.4) | -5.2 (-12.4,2.5) | -7.3 (-14.2,0.2) |  |

*Values of all models are geometric means (95% CI)*

*Abbreviations: PHDI Planetary Health Diet Index, Q quartile, CI confidence Interval, CVD cardiovascular disease, BMI Body Mass Index, GDF-15 Growth Differentiation Factor 15*

**Measured in mets*hour/week. Tertile 1:* *11.7±5.9 Tertile 2: 25.4±4.2 Tertile 3: 49.6±16.6*

Adjusted for age, sex and educational level (primary or less, secondary, university)^,^ tobacco (current, past, never smoker), alcohol consumption (never, former, moderate and heavy drinker), body mass index, energy intake, hours of TV, physical activity (mets-hour/week), diabetes and cardiovascular disease, systolic blood pressure, glucose and LDL-C levels.

**Supplementary Figure S1.** Flow diagram of included participants from the Seniors ENRICA-2 cohort.

Final sample

***N* = 2497**

Seniors ENRICA-2

2015 – 2017

***N* = 3273**

Excluded participants:

- 232 missing GDF-15.
- 483 missing/implausible energy intake.
- 1 missing educational level.
- 11 missing BMI.
- 15 missing systolic blood pressure.
- 14 missing glucose.
- 13 missing LDL-C.
- 4 missing TV hours
- 2 missing IL-6
- 1 missing hs-cTnT

*Abbreviations: BMI body mass index, GDF-15 Growth Differentiation factor 15, hs-cTnT high-sensitivity cardiac troponin T, IL-6 interleukin 6, LDL-C low-density lipoprotein cholesterol,.*
